# Telehealth *v.* face-to-face provision of care to patients with depression: a systematic review and meta-analysis

**DOI:** 10.1017/S0033291722002331

**Published:** 2022-10

**Authors:** Anna Mae Scott, Justin Clark, Hannah Greenwood, Natalia Krzyzaniak, Magnolia Cardona, Ruwani Peiris, Rebecca Sims, Paul Glasziou

**Affiliations:** Institute for Evidence-Based Healthcare, Bond University, Gold Coast, Australia

**Keywords:** Depression, meta-analysis, systematic review, telehealth, telemedicine

## Abstract

Ensuring continuity of care for patients with major depressive disorders poses multiple challenges. We conducted a systematic review and meta-analysis of randomised controlled trials comparing real-time telehealth to face-to-face therapy for individuals with depression. We searched Medline, Embase, and Cochrane Central (to November 2020), conducted a citation analysis (January 2021), and searched clinical trial registries (March 2021). We included randomised controlled trials comparing similar or identical care, delivered via real-time telehealth (phone, video) to face-to-face. Outcomes included: depression severity, quality of life, therapeutic alliance, and care satisfaction. Where data were sufficient, mean differences were calculated. Nine trials (1268 patients) were included. There were no differences between telehealth and face-to-face care for depression severity at post-treatment (SMD −0.04, 95% CI −0.21 to 0.13, *p* = 0.67) or at other time points, except at 9 months post-treatment (SMD −0.39, 95% CI −0.75 to −0.02, *p* = 0.04). One trial reported no differences in quality-of-life scores at 3- or 12-months post-treatment. One trial found no differences in therapeutic alliance at weeks 4 and 14 of treatment. There were no differences in treatment satisfaction between telehealth and face-to-face immediately post-treatment (SMD −0.14, 95% CI −0.56 to 0.28, *p* = 0.51) or at 3 or 12-months. Evidence suggests that for patients with depression or depression symptoms, the provision of care via telehealth may be a viable alternative to the provision of care face-to-face. However, additional trials are needed with longer follow-up, conducted in a wider range of settings, and with younger patients.

## Background

Depressive disorders are one of the leading causes of disability worldwide, with over 260 million adults and children affected (World Health Organization, 2020). Depressive disorders are characterised by a markedly diminished interest in usual activities and interpersonal interactions, loss of pleasure, reduced energy, and feelings of worthlessness, with these symptoms sustained over a minimum period of two weeks [American Psychiatric Association (APA), 2013]. These symptoms can lead to reduced life quality, lost productivity, and increased disability and mortality, with the global burden of depressive disorders estimated between 4.7% and 27%, with variations depending on region and tools used to determine prevalence (Ferrari et al., 2013; James et al., 2018; Wang et al., 2017).

Diagnosis of depressive disorders is largely subjective and based on clinical interviews [e.g. Structured Clinical Interview for DSM Disorder (SCID)], in addition to the use of screening instruments [such as the Patient Health Questionnaire-9 (PHQ-9), Hamilton Rating Scale (HAMD), Beck Depression Inventory II (BDI-II), Short Form Health Survey (SF-36)] which measure symptom severity and frequency (Aguilera, Ramos, Sistiva, Wang, & Alegria, 2018; Chee, Wang, & Cheung, 2020; Groth-Marnat, 2009; Serra, Spoto, Ghisi, & Vidotto, 2015; Spoto, Bottesi, Sanavio, & Vidotto, 2013). Current gold standard treatments for depressive disorders in adolescents and adults include psychological interventions such as cognitive behavioural therapy (CBT) and pharmacological treatments (American Psychiatric Association (APA), 2010; Malhi et al., 2015). For severe, chronic, or recurrent depression treatments are often combined (Petersen, 2006).

The financial costs to both individuals with depressive disorders, and societies providing care for them are projected to continue to rise in coming years (König, König, & Konnopka, 2019; Schofield et al., 2019; Wade & Häring, 2010). In addition to financial costs, many individuals find it difficult to access required care due to geographical remoteness (Moffatt & Eley, 2010). A recent review suggested that, for some health conditions, telehealth may be cost-effective and acceptable to patients (Eze, Mateus, & Hashiguchi, 2020).

As moderate and severe symptoms of depressive disorders have a significant impact on the quality of life and can lead to lost productivity and suicide, ensuring continuity of care is a priority. Telehealth has been proposed as an alternative to in-person, face-to-face care for patients living long distances from required and appropriate health services. However, previous reviews of the evidence have concluded that evidence of the effectiveness of telehealth for depression, while promising, is limited (García-Lizana & Muñoz-Mayorga, 2010; Palylyk-Colwell & Argáez, 2018).

Given the recent publication of additional trials on the effectiveness of telehealth for depression, this systematic review synthesises existing evidence from randomised controlled trials comparing the delivery of primary and/or allied healthcare interventions for depressive disorders via standard means (face-to-face) to their delivery via telehealth (e.g. video conferencing, telephone).

## Methods

This systematic review is reported following the Preferred Reporting Items for Systematic Reviews and Meta-Analyses (PRISMA) statement (Moher, Liberati, Tetzlaff, & Altman, 2009), and the review protocol was developed prospectively. Where deviations from the protocol occurred, they are reported in the appropriate methods section.

### Inclusion and exclusion criteria

We included randomised controlled trials of any design (e.g. parallel, factorial, cluster, crossover); all other study designs (observational studies, reviews, etc.) were excluded. We included studies of participants of any age or gender, who were receiving care for chronic and symptomatic depressive disorder, regardless of the severity of symptoms, whether they had received a diagnosis, duration of illness or comorbidities.

We included trials of patient care provided in primary care settings, by general practitioners, primary care/community nurses, or allied health professionals such as psychologists or counsellors, as single or multiple care visits.

Included trials compared standard care provided via telehealth (video, telephone or a combination of both), to identical or very similar care (in terms of provider, frequency, setting and duration) delivered in a face-to-face format.

We excluded trials where telehealth was provided by tertiary specialists in any setting (e.g. hospital-led telepsychiatry); mobile apps or internet-based interventions for self-management alone or in combination with telehealth modalities; interventions relying on patients entering data for real-time or delayed transmission to healthcare providers (asynchronous care); studies where novel equipment for remote monitoring was attached to patients, installed in patients' homes, or set up in a community centre; inter-professional telemedicine consultations in the absence of a patient; and interventions with multidisciplinary healthcare professionals not reflecting usual care. Trials which compared the delivery of a novel intervention for depression (rather than standard care) by telehealth to face-to-face were also excluded.

The primary outcome was depression severity, measured using any depression symptom severity scale (for example, PHQ-9, HAMD and BDI-II). Secondary outcomes included: quality of life, therapeutic alliance between the client and the care provider, and satisfaction with care (patient, caregiver and/or care provider).

### Search strategies

We conducted a search of Medline, Embase, and Cochrane CENTRAL from inception to 18 November 2020. The search string was designed for Medline and translated for use in other databases using the Polyglot Search Translator (Clark et al., 2020). This review was conducted as part of a series of systematic reviews on the effectiveness of telehealth compared to face-to-face healthcare provision in primary care or allied care for a wide range of patient groups and conditions. Therefore, the search strings were deliberately broad.

On 6 January 2021, we conducted a backwards (cited) and forwards (citing) citation analysis in Web of Science on the included studies identified by the database searches. On 25 March 2021, two clinical trial registries (clinicaltrials.gov and WHO ICTRP) were searched. Complete search strings for the databases and registry searches are provided in online Appendix 1.

No restrictions by language or publication date were imposed. We included only articles that were published in full. Abstracts for which additional details were available (e.g. a clinical registry record with results) were included; however, publications available as abstract only with no additional information were excluded.

### Study selection and screening

**Fig. 1.**
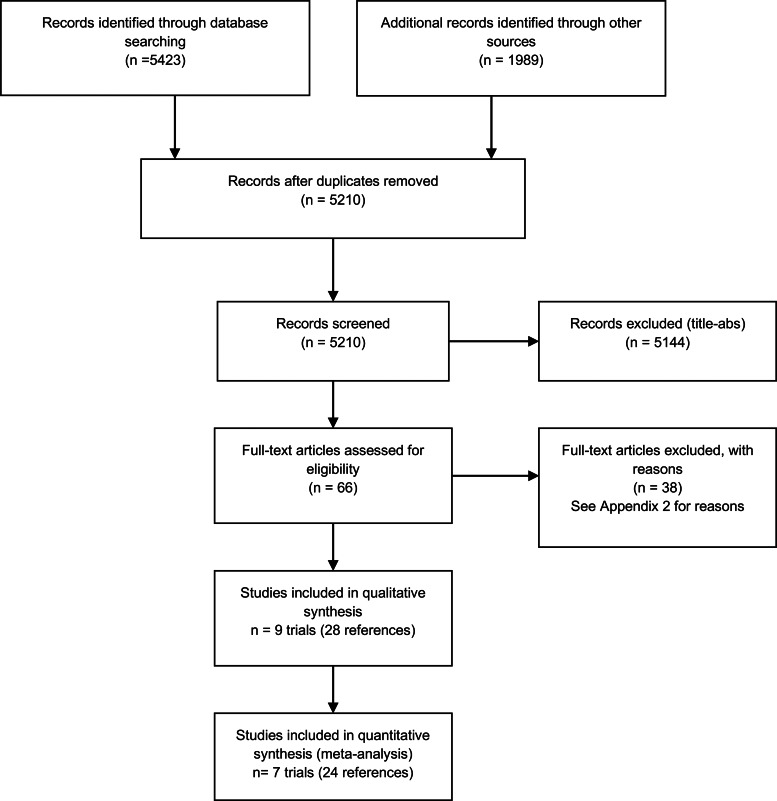
PRISMA Flow Diagram.

Paired review authors (AMS, NK, HG, MC, JC, PG, RP) independently screened titles and abstracts against the inclusion criteria. Paired review authors (AMS, HG, NK, JC, MC) retrieved full-text, and screened the full-texts for inclusion. Any disagreements were resolved by discussion, or adjudication by a third author, if required. The selection process was recorded in sufficient detail to complete a PRISMA flow diagram (Fig. 1).

### Data extraction

We extracted study characteristics (methods, participants, interventions, comparators, and outcomes), outcomes (primary and secondary) and data to inform the risk of bias judgements. Data were extracted by two authors independently (AMS, JC). Discrepancies were resolved by consensus, or adjudication by a third author, if required.

### Assessment of risk of bias in included studies

Two authors (AMS, JC) independently assessed the risk of bias for each included study using the Risk of Bias Tool 1.0, as outlined on the *Cochrane Handbook* (Higgins et al., 2019). Risk of Bias Tool 1.0 was used in preference to the Risk of Bias Tool 2.0 as the former allows the assessment of biases from conflict of interest and funding (under the domain: other sources of bias), whilst the latter does not. All disagreements were resolved by discussion or adjudication by a third author. The following domains were assessed:

1. Random sequence generation
2. Allocation concealment
3. Blinding of participants and personnel
4. Blinding of outcome assessment
5. Incomplete outcome data
6. Selective outcome reporting
7. Other bias (focusing on potential biases due to funding or conflict of interest).

Each potential source of bias was graded as low, high or unclear, and each judgement was supported by a quote from the relevant trial documented on the extraction form.

### Measurement of effect and data synthesis

*Review Manger 5.4* was used to calculate the treatment effect. For continuous outcomes (e.g. severity of illness, satisfaction measures, etc.), we used mean difference or standardised mean difference, as appropriate. We undertook meta-analyses only when meaningful (i.e. when ⩾ 2 studies or comparisons reported the same outcome). Anticipating considerable heterogeneity, we used a random effects model.

### Analyses

The individual was used as the unit of analysis, where possible. However, where data on the number of individuals with outcomes of interest was not available, we extracted the information as it was presented (e.g. mean differences between groups). We did not contact investigators or study sponsors to provide missing data.

We had intended to conduct subgroup analyses by: diagnoses within the disease category (e.g. different severities), type of healthcare provider, and time-point at which the results were reported. Due to few included trials, we did not conduct the first two subgroup analyses; however, data were sufficient to conduct subgroup analyses by time-point at which the outcome was reported for the depression severity outcome.

We had intended to conduct a sensitivity analysis by including *v.* excluding studies with 3 or more domains at high risk of bias. However, as no studies were rated at high risk of bias for 3 or more domains, we did not perform this. We had intended to conduct a sensitivity analysis by including *v.* excluding studies with fewer than 100 participants. Four studies with fewer than 100 participants were included (Glueckauf et al., 2012; Himelhoch et al., 2013; Nelson, Barnard, & Cain, 2006; Riley, Duke, Freeman, Hood, & Harris, 2015); their exclusion from the meta-analysis of the depression severity outcome did not change the estimate of effect at any time-point or overall (data not presented) – the differences between groups remain non-significant.

### Assessment of heterogeneity and reporting biases

We used the *I*^2^ statistic to measure heterogeneity among the included trials. As we did not include more than 10 trials, we did not create a funnel plot.

## Results

### Results of the search

The initial database searches yielded 5423 records, and 1989 additional records were identified through other sources – backwards (cited) and forwards (citing) analysis, as well as the clinical registry searches – for a total of 7412 records. After deduplication, there were 5210 records to screen in title and abstract. We excluded 5144 records on title and abstract, and obtained 66 records for full-text screen. We excluded 38 references on full-text screen (reasons for exclusion are provided in online Appendix 2). We included 9 trials (28 references) in the qualitative synthesis and 7 trials (24 references) in the meta-analyses (Fig. 1).

### Included studies

Nine trials (28 references), comparing telehealth to face-to-face delivery of care to patients with a depressive disorder, were includable. All trials were parallel, randomised controlled trials and all took place in the United States. Trial participants were diagnosed with or were experiencing depression symptoms, and most included only adults, except for two trials (one in children, one in youth). Evaluated therapies were cognitive and/or behaviour therapy based, including CBT (5 trials), behaviour activation (BA; 2 trials), problem-solving therapy (PST; 1 trial) and behavioural family systems therapy (BFST; 1 trial). Four trials compared therapy delivery via the telephone to its face-to-face delivery, and 5 trials compared video to face-to-face delivery. Therapy sessions ranged from 45–90 min and were generally delivered once or twice a week, for up to 18 weeks (Table 1). Follow up ranged from none (assessment immediately post-treatment) to 18 months, and trial size ranged from 14 to 325 participants. The primary and secondary outcomes were measured using a variety of scales; of the 11 scales used, 9 were self-reported, 1 was clinician reported and 1 was unclear (online Appendix 3).

**Table 1.** Characteristics of included studies

Author & Year Location	RCT design	Follow up (months)	No. participants randomised	Participants	Age years mean (s.d.)	Intervention	Care Provider	Telehealth: modality & dose	Comparator: modality & dose
Alegria 2014 (Aguilera et al., 2018; Alcántara, Li, Wang, Canino, & Alegría, 2016; Alegría et al., 2014; Kafali, Cook, Canino, & Alegria, 2014) USA	Parallel 3-arm^a^	4 months	257 (87 TH, 84 F2F, 86 usual care)^a^	Latino primary care patients with moderate or severe depressive symptoms	NR (all > 18 years old)	CBT & care management	Clinicians from various backgrounds^b^ trained in CBT	Phone Duration NR, 1x/week (1st – 4th session), 1x/2 week (sessions 5, 6) + optional 2 sessions	F2F Duration NR, 1x/week (1st – 4th session), 1x/2 week (sessions 5, 6) + optional 2 sessions
Choi 2014 (Namkee G. Choi et al., 2014a; Namkee G. Choi, Hegel, Sirrianni, Marinucci, & Bruce, 2012; N. G. Choi et al., 2014b; N. G. Choi, Marti, & Conwell, 2016) USA	Parallel 3-arm^c^	18 months	158 (56 TH, 63 F2F, 39 care calls)^c^	Low-income homebound older adults with depression	65 (s.d. 9)	BST	Master's level social workers	Video 60 min, 1st session F2F + 5 sessions by video^d^	F2F 60 min, 6 sessions F2F
Egede 2015(Egede et al., 2015; Egede et al., 2016; L. E. Egede, Dismuke, Walker, Acierno, & Frueh, 2018; L. E. Egede et al., 2017) USA	Parallel 2-arm	12 months	241 (120 TH, 121 F2F)	Veterans >58yo, with a major depressive disorder	64 (s.d. 5)	BA	Master's level counsellors with 5+ years' experience	Video 60 min, 1x/week, 8 weeks	F2F 60 min, 1x/week, 8 weeks
Glueckauf 2012 (Glueckauf et al., 2012; Meng et al., 2021) USA	Parallel 2-arm	N/A^e^	14 (7 TH, 7 F2F)	African American dementia caregivers with depression	58 (s.d. 10)	CBT	Master's level counsellors	Phone 60 min, 1x/week, 12 weeks	F2F 60 min, 1x/week, 12 weeks
Himmelhoch 2013(Himelhoch et al., 2013) USA	Parallel 2-arm	N/A^e^	34 (16 TH, 18 F2F)	Urban, low-income people with HIV/AIDS & depression	45 (s.d. 8)	CBT	Master's level therapists	Phone 45 min, 11 sessions over 14 weeks	F2F 45 min, 11 sessions over 14 weeks
Luxton 2016 (Bounthavong et al., 2018; Luxton et al., 2016; Pruitt et al., 2018; Smolenski, Pruitt, Vuletic, Luxton, & Gahm, 2017) USA	Parallel 2-arm	3 months	121 (62 TH, 59 F2F)	US Military Personnel and Veterans with depression	NR (range 19–65)	BA treatment for depression	Doctorate-level mental health providers	Video 50–60 min, 1x/week, 8 weeks	F2F 50–60 min, 1x/week, 8 weeks
Mohr 2012 (Kalapatapu et al., 2014; Mohr et al., 2012; Stiles-Shields, Corden, Kwasny, Schueller, & Mohr, 2015; Stiles-Shields, Kwasny, Cai, & Mohr, 2014a, 2014b) USA	Parallel 2-arm	6 months	325 (163 TH, 162 F2F)	Primary care patients with major depressive disorder	48 (s.d. 13)	CBT	PhD level psychologists	Phone 45 min, 2x/week (session 1–4), 1x/week (5–16), 2x/2 week (17, 18); 18 weeks total	F2F 45 min, 2x/week (session 1–4), 1x/ week (5–16), 2x/2 week (17, 18); 18 weeks total
Nelson 2006 (Nelson et al., 2006; E. L. Nelson, Barnard, & Cain, 2003) USA	Parallel 2-arm	N/A^e^	28 (14 TH, 14 F2F)	Children with depression	NR (range 8–14)	CBT	CBT therapist	Video 90 min (1st session), 60 min (others); 1x/week, 8 weeks	F2F 90 min (1st session), 60 min (others); 1x/week, 8 weeks
Riley 2015 (Riley, Duke, Freeman, Hood, and Harris, 2015) USA	Parallel 2-arm	3 months	90 (46 TH, 44 F2F)	Youth with T1D > 1 year w suboptimal glycaemic control, and depressive symptoms	15 (s.d. 2)	BFST diabetes	Masters or doctorate level clinical psychologist	Video 60–90 min, up to 10 sessions over a 12 week period	F2F 60–90 min, up to 10 sessions over a 12 week period

T1D, type 1 diabetes; TH, telehealth; F2F, face to face; CBT, cognitive behavioural therapy; BA, behavioural activation; PST, problem-solving therapy; BFST, behavioural family systems therapy.

a Usual care arm was excluded from the present analysis.

b Includes 3 clinical psychologists.

c 3rd arm (care calls) excluded from the present analysis.

d Timeframe for treatment not reported; 2 social workers and 1 counsellor.

e Assessed immediately post-intervention (no follow-up).

### Risk of bias

**Fig. 2.**
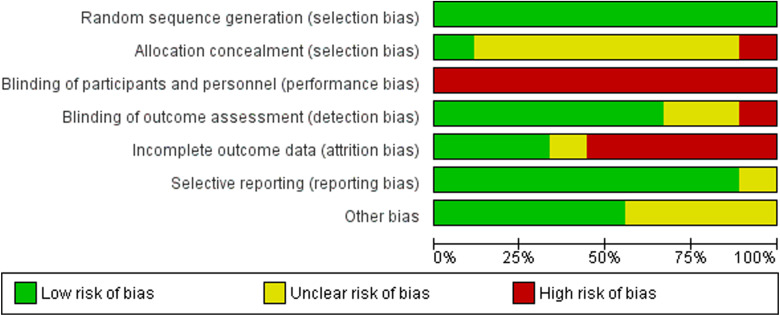
Risk of bias graph: review authors' judgements about each risk of bias item presented as percentages across all included studies.

Overall, the risk of bias for the included trials was generally low or unclear, except for blinding and incomplete outcome data. Risk of bias was low for random sequence generation, and most studies were rated at unclear risk of bias from allocation concealment (mainly due to non-reporting). All trials were at high risk of bias for blinding of participants and personnel, as the nature of the compared interventions (video or telephone *v.* face-to-face delivery of care) rendered patient blinding impossible. More than half the trials were at high risk of attrition bias, due to the high attrition of participants from the trial. The risk of reporting bias and other bias (due to funding and conflict of interest) were generally low or unclear, mainly due to the lack of reporting (Fig. 2).

### Effectiveness of the intervention

#### Primary outcome: depression severity

**Fig. 3.**
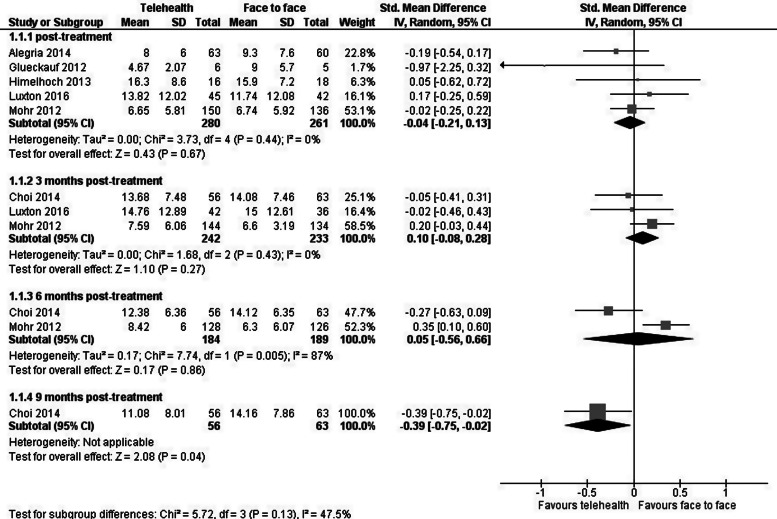
Telehealth *v.* face-to-face care for patients with depression: impact on the depression severity outcome.

Nine trials reported on the effect of treatment on depression severity, the results from 6 of which were meta-analysable. There were no statistically or clinically significant differences between telehealth and face-to-face trial arms for depression severity immediately (SMD −0.04, 95% CI −0.21 to 0.13, *p* = 0.67), at 3 months (SMD 0.10, 95% CI −0.08 to 0.28, *p* = 0.27), or at 6 months post-treatment (SMD 0.05, 95% CI −0.56 to 0.66, *p* = 0.86). There was a significant difference (favouring telehealth) from the 1 trial with results at 9 months post-treatment (SMD −0.39, 95% CI −0.75 to −0.02, *p* = 0.04). Heterogeneity immediately post-treatment and at 3 months post-treatment was very low (*I*^2^ = 0%); it was 87% at 6 months post-treatment (Fig. 3).

Three included trials were not meta-analysable. One trial reported a non-significant mean difference between telehealth and face-to-face in BDI scores at 3 months (MD −3.72%, 90% CI −13.86% to 6.41%) and at 12 months (1.05%, 90% CI −8.3% to 10.41%) post-intervention (Egede et al., 2015). One trial reported the mean Children's Depression Inventory (CDI) score change for the telehealth and face-to-face care recipients combined, reporting a score change from a mean of 13.96 (s.d. 9.15) prior to the intervention to 9.18 (s.d. 9.08) post-intervention; 82% of participants had remission (as operationalised by study authors) from depression immediately post-treatment, with similar rates observed for both face-to-face and telehealth care (Nelson et al., 2006). Another trial similarly reported a change in CDI scores pre- to post-treatment, with a significant decrease in CDI scores from pre- to immediately post-treatment (*p* ⩽ 0.05) and from pre- to 3 months post-treatment (*p* ⩽ 0.001) (Riley et al., 2015).

#### Secondary outcome: quality of life score

Only one trial reported on 3- and 12-month post-treatment quality of life outcomes (Egede et al., 2016). There were no significant differences between the telehealth and the face-to-face care at either 3- or 12-months post-treatment in SF-36 scores, in any of the assessed domains (physical function, limits due to physical health, limits due to emotional problems, energy/vitality, emotional well-being, social functioning, pain, general health).

#### Secondary outcome: therapeutic alliance

Only one trial reported on the therapeutic alliance (Mohr et al., 2012; Stiles-Shields, Kwasny, Cai, & Mohr, 2014b) measured using the Working Alliance Inventory Short Form patient version (WAI-C) and therapist version (WAI-T) at weeks 4 and 14 during the trial. For therapists, there was no significant difference in the WAI-T score between the telehealth and face-to-face care conditions either at week 4 (MD −0.03, 95% CI −2.02 to 1.97, *p* = 0.98) or week 14 (MD 0.61, 95% CI −1.26 to 2.48, *p* = 0.52). Similarly, for patients there were no differences between telehealth and face-to-face care, either at week 4 (MD 0.21 95% CI −1.27 to 1.68, *p* = 0.78) or week 14 (MD 0.77, 95% CI −0.84 to 2.37, *p* = 0.35).

#### Secondary outcome: treatment satisfaction

**Fig. 4.**
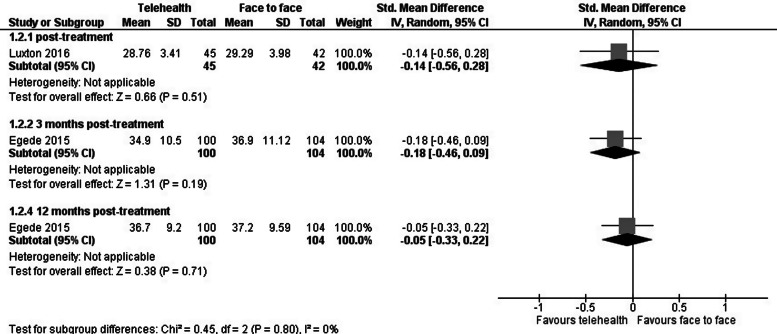
Telehealth *v.* face-to-face care for patients with depression: impact on treatment satisfaction.

Of the three trials that reported on treatment satisfaction, 2 were meta-analysable. There were no differences in treatment satisfaction between the telehealth and face-to-face care conditions immediately (*p* = 0.51), at 3 months (*p* = 0.19) or at 12 months post-treatment (*p* = 0.71) (Fig. 4).

One trial reported on the satisfaction of the children and their parents who completed a telemedicine satisfaction questionnaire (14 parents and 14 children). Thirteen of the 14 parents and all 14 children agreed with the statement that telemedicine is ‘as good as face-to-face’ (Nelson et al., 2006).

## Discussion

This systematic review of 9 trials (which included 1268 patients in aggregate) found evidence that psychological interventions delivered via telehealth or delivered face-to-face lead to similar outcomes for depressive symptom severity, quality of life, therapeutic alliance, and treatment satisfaction in both adults and young people. The included trials were generally at low risk of bias (excepting the risk of bias from blinding of the participants, which was not possible due to the nature of the compared interventions).

Our review identified several evidence gaps. First, trial follow-up was generally short (6 of the 9 trials followed up patients for 4 months or less). As depressive disorders are frequently considered chronic, long-term conditions [American Psychological Association (APA), 2019], this presents uncertainty of the long-term effectiveness of telehealth *v.* face-to-face delivered intervention for depressive disorders. Further, comorbidities and concurrent pharmacological treatment of trial participants are largely unknown, potentially impacting patient prognosis and the efficacy, appropriateness, and satisfaction with telehealth services (Steffen, Nübel, Jacobi, Bätzing, & Holstiege, 2020).

Second, all trials were conducted in the United States. Healthcare system in the USA may not be comparable to those elsewhere (e.g. Australia, Canada, UK) (Schütte, Acevedo, & Flahault, 2018), which may limit the generalisability of the findings to other countries and medical systems. In addition to healthcare systems, geographical location (e.g. rural, remote) has been reported to influence patients' ease of accessing required healthcare, partially due to limited accessibility of appropriate healthcare services and significant travel requirements to access these (Moffatt & Eley, 2010). Telehealth may present an opportunity for increasing accessibility in these populations; however, further analysis of telehealth by location (e.g. regional, metropolitan) is required to determine whether telehealth efficacy is consistent across locations (Bradford, Caffery, & Smith, 2015). While the included studies provided information regarding the type of telehealth utilised (e.g. telephone, video), additional research into the specific platforms (e.g. Zoom, Coviu) utilised to provide telehealth may be beneficial in determining potential differences in usability, functionality, and patient satisfaction.

Third, while two of the nine included trials were conducted in participants under 18 years of age, it is unclear whether any of the remaining trials were conducted in the elderly, limiting the generalisability of findings to the latter population. Depressive disorders also occur in the elderly and demonstrate similar financial and emotional burdens to depressive disorders that occur in other age groups (Fiske, Wetherell, & Gatz, 2009). Therefore, it will be important to further examine the effectiveness of telehealth in elderly patients with depressive disorders, particularly as ease of access and use of telehealth may be compromised in this population (Gentry, Lapid, & Rummans, 2019).

Finally, the evidence for the outcomes of quality of life and therapeutic alliance is limited to one trial each, necessitating further studies to confirm the findings. Quantitative examination of these, including ease of use, patient engagement, and work burden, is necessary to determine the potential barriers to the successful use of telehealth, both for depressive disorders and health consultations more widely (MacNeill et al., 2014).

This review's strengths include its comprehensive searches and rigorous methodologies. The included trials examined several psychological cognitive and/or behavioural interventions which utilised crucial elements of current gold standard treatments for depressive disorders. The findings of our review support previous review findings, which suggest that adults completing telehealth, compared to face-to-face, intervention for depression report equal or significantly higher satisfaction with services, and no statistically significant differences in symptom improvement (Guaiana, Mastrangelo, Hendrikx, & Barbui, 2021). Further, encompassing depressive symptomology generally, as opposed to specific disorder diagnoses (e.g. major depressive disorder), allows for wider generalisability of findings beyond those clinically diagnosed to those experiencing undiagnosed depression or depression symptoms.

Telehealth – via telephone or video – has the potential to increase the accessibility of effective, evidence-based interventions for depressive disorders in patients facing geographical or logistical challenges in attending face-to-face interventions. Additional research to increase confidence in the comparability of telehealth and face-to-face intervention for depressive disorders, including in varied populations and locations, with longer follow-up, and measuring key outcomes of importance to both the patients and the clinicians, is warranted. However, the results of this review suggest, particularly in the short-term, that telehealth may present a feasible alternative to face-to-face intervention for individuals with depressive disorders.

## Supplementary material

For supplementary material accompanying this paper visit https://doi.org/10.1017/S0033291722002331. click here to view supplementary material
