# Supplementary material for: Telehealth v. face-to-face provision of care to patients with depression: a systematic review and meta-analysis
Source: Psychol Med. 2022 Aug 12;52(14):2852–60. doi: 10.1017/S0033291722002331 (PMC9693715; doi:10.1017/S0033291722002331)
Supplement: Supplementary file 1 [file S0033291722002331sup001.docx]

# Appendix 1: Searches

## Database searches

**PubMed Search run 18/11/2020**

("Telemedicine"[Mesh] OR "Videoconferencing"[Mesh] OR Telehealth[tiab] OR Telemedicine[tiab] OR Videoconferencing[tiab] OR ((Telephone[tiab]) AND (Consultation[tiab] OR face-to-face[tiab] OR in-person[tiab])) OR telephone-delivered[tiab])

AND

("Primary Health Care"[Mesh] OR "General Practice"[Mesh] OR rehabilitation[sh] OR "Outpatients"[Mesh] OR "Speech Therapy"[Mesh] OR Outpatient[tiab] OR “Primary health”[tiab] OR “Primary care”[tiab] OR “General practice”[tiab] OR “General practices”[tiab] OR “General practitioners”[tiab] OR “General practitioner”[tiab] OR “Family practice”[tiab] OR Physician[tiab] OR Physicians[tiab] OR Clinician[tiab] OR Clinicians[tiab] OR Therapist[tiab] OR Nurse[tiab] OR Nurses[tiab] OR Physiotherapist[tiab] OR Rehabilitation[tiab] OR Diabetes[tiab] OR Diabetic[tiab] OR Asthma[tiab] OR Depression[tiab] OR “Ïrritable bowel”[tiab] OR IBS[tiab] OR PTSD[tiab] OR “Chronic fatigue”[tiab])

AND

((Face-to-face[tiab]) OR “Usual care”[tiab] OR Visits[tiab] OR Visit[tiab] OR In-person[tiab] OR “In person”[tiab] OR ((Clinic[tiab] OR Centre[tiab] OR Home[tiab]) AND (Based[tiab] OR Contact[tiab])) OR Conventional[tiab] OR “Practice-based”[tiab] OR “Practice based”[tiab] OR Traditional[tiab] OR “Standard care”[tiab] OR Homecare[tiab] OR ((Routine[tiab] OR Home[tiab]) AND (Care[tiab])))

AND

("Delivery of Health Care"[Mesh] OR Delivery[tiab] OR Delivered[tiab] OR Via[tiab] OR Received[tiab])

AND

("Treatment Outcome"[Mesh] OR "Patient Satisfaction"[Mesh] OR Therapy[sh] OR Diagnosis[sh] OR “Clinical outcomes”[tiab] OR Treatment[tiab] OR Diagnostic[tiab] OR Efficacy[tiab])

AND

(Randomized controlled trial[pt] OR controlled clinical trial[pt] OR randomized[tiab] OR randomised[tiab] OR placebo[tiab] OR "drug therapy"[sh] OR randomly[tiab] OR trial[tiab] OR groups[tiab]) NOT

(Animals[Mesh] not (Animals[Mesh] and Humans[Mesh]))

NOT

(“Case Reports”[pt] OR Editorial[pt] OR Letter[pt] OR Meta-Analysis[pt] OR “Observational Study”[pt] OR “Systematic Review”[pt] OR “Case Report”[ti] OR “Case series”[ti] OR Meta-Analysis[ti] OR “Meta Analysis”[ti] OR “Systematic Review”[ti] OR “Systematic Literature Review”[ti] OR “Qualitative study”[ti] OR Protocol[ti])

**CENTRAL via the Cochrane Library run 18/11/2020**

([mh Telemedicine] OR [mh Videoconferencing] OR Telehealth:ti,ab OR Telemedicine:ti,ab OR Videoconferencing:ti,ab OR ((Telephone:ti,ab) AND (Consultation:ti,ab OR “ face-to-face”:ti,ab OR “in person”:ti,ab)) OR “telephone delivered”:ti,ab)

AND

([mh "Primary Health Care"] OR [mh "General Practice"] OR [mh Outpatients] OR [mh "Speech Therapy"] OR Outpatient:ti,ab OR "Primary health":ti,ab OR "Primary care":ti,ab OR "General practice":ti,ab OR "General practices":ti,ab OR "General practitioners":ti,ab OR "General practitioner":ti,ab OR "Family practice":ti,ab OR Physician:ti,ab OR Physicians:ti,ab OR Clinician:ti,ab OR Clinicians:ti,ab OR Therapist:ti,ab OR Nurse:ti,ab OR Nurses:ti,ab OR

Physiotherapist:ti,ab OR Rehabilitation:ti,ab OR Diabetes:ti,ab OR Diabetic:ti,ab OR Asthma:ti,ab OR Depression:ti,ab OR "Ïrritable bowel":ti,ab OR IBS:ti,ab OR PTSD:ti,ab OR "Chronic fatigue":ti,ab)

AND

(("Face-to-face":ti,ab) OR "Usual care":ti,ab OR Visits:ti,ab OR Visit:ti,ab OR "In person":ti,ab OR ((Clinic:ti,ab OR Centre:ti,ab OR Home:ti,ab) AND (Based:ti,ab OR Contact:ti,ab)) OR Conventional:ti,ab OR "Practice based":ti,ab OR Traditional:ti,ab OR "Standard care":ti,ab OR Homecare:ti,ab OR ((Routine:ti,ab OR Home:ti,ab) AND (Care:ti,ab)))

AND

([mh "Delivery of Health Care"] OR Delivery:ti,ab OR Delivered:ti,ab OR Via:ti,ab OR Received:ti,ab)

AND

([mh "Treatment Outcome"] OR [mh "Patient Satisfaction"] OR "Clinical outcomes":ti,ab OR Treatment:ti,ab OR Diagnostic:ti,ab OR Efficacy:ti,ab)

**Embase search run 18/11/2020**

('Telemedicine'/exp OR 'Videoconferencing'/exp OR Telehealth:ti,ab OR Telemedicine:ti,ab OR Videoconferencing:ti,ab OR ((Telephone:ti,ab) AND (Consultation:ti,ab OR face-to-face:ti,ab OR in-person:ti,ab)) OR telephone-delivered:ti,ab)

AND

('Primary Health Care'/exp OR 'General Practice'/exp OR 'Outpatient'/exp OR 'Speech Therapy'/exp OR Outpatient:ti,ab OR "Primary health":ti,ab OR "Primary care":ti,ab OR "General practice":ti,ab OR "General practices":ti,ab OR "General practitioners":ti,ab OR "General practitioner":ti,ab OR "Family practice":ti,ab OR Physician:ti,ab OR Physicians:ti,ab OR Clinician:ti,ab OR Clinicians:ti,ab OR Therapist:ti,ab OR Nurse:ti,ab OR Nurses:ti,ab OR Physiotherapist:ti,ab OR Rehabilitation:ti,ab OR Diabetes:ti,ab OR Diabetic:ti,ab OR Asthma:ti,ab OR Depression:ti,ab OR "Ïrritable bowel":ti,ab OR IBS:ti,ab OR PTSD:ti,ab OR "Chronic fatigue":ti,ab)

AND

(("Face-to-face":ti,ab) OR "Usual care":ti,ab OR Visits:ti,ab OR Visit:ti,ab OR In-person:ti,ab OR "In person":ti,ab OR ((Clinic:ti,ab OR Centre:ti,ab OR Home:ti,ab) AND (Based:ti,ab OR Contact:ti,ab)) OR Conventional:ti,ab OR Practice-based:ti,ab OR "Practice based":ti,ab OR Traditional:ti,ab OR "Standard care":ti,ab OR Homecare:ti,ab OR ((Routine:ti,ab OR Home:ti,ab) AND (Care:ti,ab)))

AND

('health care delivery'/exp OR Delivery:ti,ab OR Delivered:ti,ab OR Via:ti,ab OR Received:ti,ab)

AND

('Treatment Outcome'/exp OR 'Patient Satisfaction'/exp OR "Clinical outcomes":ti,ab OR Treatment:ti,ab OR Diagnostic:ti,ab OR Efficacy:ti,ab)

AND

(random* OR factorial OR crossover OR placebo OR blind OR blinded OR assign OR assigned OR allocate OR allocated OR 'crossover procedure'/exp OR 'double-blind procedure'/exp OR 'randomized controlled trial'/exp OR 'single-blind procedure'/exp NOT ('animal'/exp NOT ('animal'/exp AND 'human'/exp)))

AND [embase]/lim

## Clinical registry searches

Searches run 25/03/2021

**Telehealth and depression**

Clinicaltrials.gov

Intervention field: (Telemedicine OR Videoconferencing OR Telephone OR Telehealth) AND (“Usual care” OR “Standard care” OR Face-to-face OR Face to face”)

Condition or disease field: Depression

WHO ICTRP

Telemedicine AND Depression OR Telehealth AND Depression OR Videoconferencing AND Depression

# Appendix 2: Table of Excluded Studies

| **No.** | **Reference** | **Reason for exclusion** |
| --- | --- | --- |
| 1 | Aburizik A, Dindo L, Kaboli P, Charlton M, Dawn K, Turvey C. A pilot randomized controlled trial of a depression and disease management program delivered by phone. J Affect Disord. 2013;151(2):769-74. | Care not comparable between groups |
| 2 | Aguilera A, Ramos Z, Sistiva D, Wang Y, Alegria M. Homework Completion via Telephone and In-Person Cognitive Behavioral Therapy Among Latinos. Cognitive Therapy and Research. 2018;42(3):340-7. | Duplicate |
| 3 | Baumrucker SJ. Telephone-based versus face-to-face cognitive behavioral therapy for depression. Journal of Pain and Symptom Management. 2012;44(5):794. | Study type |
| 4 | Bounthavong M, Pruitt LD, Smolenski DJ, Gahm GA, Bansal A, Hansen RN. Economic evaluation of in-home telehealth compared to in-person treatment delivery for managing depression. Value in Health. 2016;19(3):A189. | Conference abstract for an included full-text |
| 5 | Choi NG, Hegel MT, Nathan Marti C, Lynn Marinucci M, Sirrianni L, Bruce ML. Telehealth problem-solving therapy for depressed low-income homebound older adults. American Journal of Geriatric Psychiatry. 2012. | Duplicate |
| 6 | Deen TL, Fortney JC, Schroeder G. Patient Acceptance of and Initiation and Engagement in Telepsychotherapy in Primary Care. Psychiatric Services. 2013;64(4):380-4. | Study type |
| 7 | Deitsch SE, Frueh BC, Santos AB. Telepsychiatry for post-traumatic stress disorder. Journal of Telemedicine and Telecare. 2000;6(3):184-6. | Study type |
| 8 | Dietrich AJ, Oxman TE, Williams JW, Jr., Schulberg HC, Bruce ML, Lee PW, et al. Re-engineering systems for the treatment of depression in primary care: cluster randomised controlled trial. Bmj. 2004;329(7466):602. | Care not comparable between groups |
| 9 | Dixon P, Hollinghurst S, Edwards L, Thomas C, Foster A, Davies B, et al. Cost-effectiveness of telehealth for patients with depression: evidence from the Healthlines randomised controlled trial. Bjpsych Open. 2016;2(4). | Comparator |
| 10 | Donohue JM, Belnap BH, Men A, He F, Roberts MS, Rollman BL. 12-Month cost-effectiveness of telephonedelivered collaborative care for treating post-CABG depression. Psychosomatic Medicine. 2012;74(3):A91. | Care not comparable between groups |
| 11 | Dwight-Johnson M, Aisenberg E, Golinelli D, Hong S, O'Brien M, Ludman E. Telephone-based cognitive-behavioral therapy for Latino patients living in rural areas: a randomized pilot study. Psychiatr Serv. 2011;62(8):936-42. | Care not comparable between groups |
| 12 | Egede LE, Walker RJ, Payne EH, Knapp RG, Acierno R, Frueh BC. Effect of psychotherapy for depression via home telehealth on glycemic control in adults with type 2 diabetes: Subgroup analysis of a randomized clinical trial. Journal of Telemedicine and Telecare. 2018;24(9):596-602. | Population |
| 13 | Fann JR, Bombardier CH, Vannoy S, Dyer J, Ludman E, Dikmen S, et al. Telephone and in-person cognitive behavioral therapy for major depression after traumatic brain injury: a randomized controlled trial. J Neurotrauma. 2015;32(1):45-57. | Study type |
| 14 | Germain V, Marchand A, Bouchard S, Drouin M-S, Guay S. Effectiveness of Cognitive Behavioural Therapy Administered by Videoconference for Posttraumatic Stress Disorder. Cognitive Behaviour Therapy. 2009;38(1):42-53. | Study type |
| 15 | Gros DF, Yoder M, Tuerk PW, Lozano BE, Acierno R. Exposure Therapy for PTSD Delivered to Veterans via Telehealth: Predictors of Treatment Completion and Outcome and Comparison to Treatment Delivered in Person. Behavior Therapy. 2011;42(2):276-83. | Study type |
| 16 | Heckman TG, Heckman BD, Anderson T, Lovejoy TI, Markowitz JC, Shen Y, et al. Tele-Interpersonal Psychotherapy Acutely Reduces Depressive Symptoms in Depressed HIV-Infected Rural Persons: A Randomized Clinical Trial. Behav Med. 2017;43(4):285-95. | Care not comparable between groups |
| 17 | Heckman TG, Markowitz JC, Heckman BD, Woldu H, Anderson T, Lovejoy TI, et al. A Randomized Clinical Trial Showing Persisting Reductions in Depressive Symptoms in HIV-Infected Rural Adults Following Brief Telephone-Administered Interpersonal Psychotherapy. Annals of Behavioral Medicine. 2018;52(4):299-308. | Comparator |
| 18 | Hudson TJ, Fortney JC, Lu L, Pyne JM, Mittal D. Comparison of antidepressant side effects in telemedicine-based versus practice-based collaborative care. Clinical and Translational Science. 2012;5(2):198. | Care not comparable between groups |
| 19 | Kirkness CJ, Becker KJ, Cain KC, Kohen R, Tirschwell DL, Teri L, et al. Telephone versus in-person psychosocial behavioral treatment in post-stroke depression. Stroke. 2015;46. | Study of a novel intervention |
| 20 | Kirkness CJ, Cain KC, Becker KJ, Tirschwell DL, Buzaitis AM, Weisman PL, et al. Randomized trial of telephone versus in-person delivery of a brief psychosocial intervention in post-stroke depression. BMC Res Notes. 2017;10(1):500. | Study of a novel intervention |
| 21 | Kooistra L, Ruwaard J, Wiersma J, van Oppen P, Riper H. Working Alliance in Blended Versus Face-to-Face Cognitive Behavioral Treatment for Patients with Depression in Specialized Mental Health Care. Journal of Clinical Medicine. 2020;9(2). | Comparator |
| 22 | Luxton DD, Pruitt LD, Wagner A, Smolenski DJ, Jenkins-Guarnieri MA, Gahm G. Home-Based Telebehavioral Health for US Military Personnel and Veterans With Depression: A Randomized Controlled Trial. Journal of Consulting and Clinical Psychology. 2016;84(11):923-34. | Duplicate |
| 23 | Mitchell JE, Crosby RD, Wonderlich SA, Crow S, Lancaster K, Simonich H, et al. Randomized trial comparing the efficacy of cognitive-behavioral therapy for bulimia nervosa delivered via telemedicine versus face-to-face. Behaviour Research and Therapy. 2008;46(5):581-92. | Population |
| 24 | Mittal D, Chekuri L, Lu L, Fortney JC. Demographic, economic, and clinical correlates of depression treatment response in an underserved primary care population. Journal of Clinical Psychiatry. 2014;75(8):848-54. | Care not comparable between groups |
| 25 | Preschl B, Maercker A, Wagner B. The working alliance in a randomized controlled trial comparing online with face-to-face cognitive-behavioral therapy for depression. BMC Psychiatry. 2011;11:189. | Intervention |
| 26 | Pyne JM, Fortney JC, Mouden S, Lu L, Hudson TJ, Mittal D. Cost-effectiveness of on-site versus off-site collaborative care for depression in rural FQHCs. Psychiatr Serv. 2015;66(5):491-9. | Care not comparable between groups |
| 27 | Pyne JM, Fortney JC, Mouden S, Lu L, Hudson TJ, Mittal D. Cost-Effectiveness of On-Site Versus Off-Site Collaborative Care for Depression in Rural FQHCs. Psychiatric Services. 2015;66(5):491-9. | Care not comparable between groups |
| 28 | Pyne JM, Fortney JC, Tripathi SP, Maciejewski ML, Edlund MJ, Williams DK. Cost-effectiveness Analysis of a Rural Telemedicine Collaborative Care Intervention for Depression. Archives of General Psychiatry. 2010;67(8):812-21. | Care not comparable between groups |
| 29 | Reed M. Telephonic CBT versus face-to-face CBT for depression. 2012. | Study type |
| 30 | Reese RJ, Mecham MR, Vasilj I, Lengerich AJ, Brown HM, Simpson NB, et al. The effects of telepsychology format on empathic accuracy and the therapeutic alliance: An analogue counselling session. Counselling & Psychotherapy Research. 2016;16(4):256-65. | Population |
| 31 | Sarkar S, Gupta R. Telephone vs Face-to-Face Cognitive Behavioral Therapy for Depression. Jama-Journal of the American Medical Association. 2012;308(11):1090-1. | Study type |
| 32 | Shulman M, John M, Kane JM. Home-Based Outpatient Telepsychiatry to Improve Adherence With Treatment Appointments: A Pilot Study. Psychiatric Services. 2017;68(7):743-6. | Population |
| 33 | Stewart RW, Orengo-Aguayo R, Young J, Wallace MM, Cohen JA, Mannarino AP, et al. Feasibility and Effectiveness of a Telehealth Service Delivery Model for Treating Childhood Posttraumatic Stress: A Community-Based, Open Pilot Trial of Trauma-Focused Cognitive-Behavioral Therapy. Journal of Psychotherapy Integration. 2020;30(2):274-89. | Study type |
| 34 | Stewart RW, Orengo-Aguayo RE, Cohen JA, Mannarino AP, de Arellano MA. A Pilot Study of Trauma-Focused Cognitive-Behavioral Therapy Delivered via Telehealth Technology. Child Maltreatment. 2017;22(4):324-33. | Study type |
| 35 | Temkin NR, Fann J, Bombardier C, Vannoy S, Dikmen S, Ludman E. Telephone and in-person cognitive behavioral therapy for depression after traumatic brain injury. Journal of Neurotrauma. 2012;29(10):A96-A7. | Study type |
| 36 | Thase ME, McCrone P, Barrett MS, Eells TD, Wisniewski SR, Balasubramani GK, et al. Improving Cost-effectiveness and Access to Cognitive Behavior Therapy for Depression: Providing Remote-Ready, Computer-Assisted Psychotherapy in Times of Crisis and Beyond. Psychotherapy and Psychosomatics. 2020;89(5):307-13. | Care not comparable between groups |
| 37 | Thompson DA, Leimig R, Gower G, Winsett RP. Assessment of depressive symptoms during post-transplant follow-up care performed via telehealth. Telemed J E Health. 2009;15(7):700-6. | Care not comparable between groups |
| 38 | Wagner B, Horn AB, Maercker A. Internet-based versus face-to-face cognitive-behavioral intervention for depression: A randomized controlled non-inferiority trial. Journal of Affective Disorders. 2014;152:113-21. | Intervention |

# Appendix 3: Table of Scales used to measure outcomes

| **Outcome** | **Acronym** | **Full Name** | **Study** | **Use of scale** | **Quality** | **Items** | **Delivery** | **Scoring** |
| --- | --- | --- | --- | --- | --- | --- | --- | --- |
| Depression Severity | PHQ-9 | Patient Health Questionnaire | Alegria 2014, Mohr 2012 | used in screening for probable depression and monitoring treatment progress | reliable and valid | 9 | Self-report | Total score of 27, higher score indicates more severe depression symptoms |
|  | CES-D | Center for Epidemiological Studies Survey-Depression scale | Glueckauf 2012 | assesses depression in nonclinical community populations | reliable and valid | 20 | Self-report | A total score ranging from 0 to 60 is derived by summing the item scores. Individuals scoring 16 or higher on the CES-D are generally considered to be at risk for developing clinical depression. |
|  | HAMD | Hamilton Rating Scale for Depression | Choi 2012, Himelhoch 2013 | designed for adults and is used to rate the severity of their depression | reliable and valid | 17 | Clinician rated | Scored to get a total score, higher the total score the more severe the depression |
|  | BDI/  BDI-II | Beck Depression Inventory | Egede 2015*, Luxton 2016 | evaluating the severity of depression in normal and psychiatric populations | reliable and valid | 21 | Self-report | Sum scores are calculated with a possible range of 0 to 63, with higher scores indicating higher levels of depression symptom severity |
|  | CDI | Children's depression inventory | Nelson 2006*, Riley 2015 * | to capture the child’s subjective experience of his or her symptoms | reliable and valid | 27 | Self-report | Higher scores indicate more severe depressive symptoms, with scores ranging from 0 to 54 |
| Quality of Life | SF-36 | Short Form Survey | Egede, 2016* | Measures health status and functioning over the past 4 weeks | Reliable and valid | 26 | Self-report | Final scores range from 0-100, with the highest level of functioning being 100 |
| Therapeutic working alliance | WAI-T | Working Alliance Inventory Short Form - Therapist version | Mohr 2012 (in Stiles-Shield, 2014)* | refined measure of the therapeutic alliance that assesses three key aspects of the therapeutic alliance | Reliable and valid | 12 | Self-report | Higher score reflect more positive working alliance, scored on a scale of 1-7 |
|  | WAI-C | Working Alliance Inventory Short Form - Client version | Mohr 2012 (in Stiles-Shield, 2014)* | refined measure of the therapeutic alliance that assesses three key aspects of the therapeutic alliance | Reliable and valid | 12 | Self-report | Higher score reflect more positive working alliance, scored on a scale of 1-7 |
| Satisfaction | CSQ-8 | Client Satisfaction Questionnaire | Luxton 2016 | measure of general satisfaction with psychotherapeutic treatment | reliable and valid | 8 | Self-report | Participants are asked to rate satisfaction on a 4-point scale, with a possible range of 8 to 32, with higher scores indicating greater satisfaction |
|  | CPOSS | Charleston Psychiatrics Outpatient Satisfaction Scale | Egede 2015 (in Egede 2016) | used to measure satisfaction in psychiatric outpatients | Reliable and valid | 16 | Self-report | The overall score results from summing responses to individiual questions for a possible range of 13 to 65 with higher scores indicating higher satisfaction |
|  | TSQ | Telemedicine Satisfaction Questionnaire | Nelson, 2003* | measures satisfaction with the telemedicine consult | Unclear | Unclear | Unclear | Unclear |

* not included in meta-analysis
